# Household air pollution and the sustainable development goals

**DOI:** 10.2471/BLT.15.155812

**Published:** 2016-01-21

**Authors:** Adeladza Kofi Amegah, Jouni JK Jaakkola

**Affiliations:** a Department of Biomedical Sciences, School of Allied Health Sciences, University of Cape Coast, University Avenue, Cape Coast, Ghana.; b Center for Environmental and Respiratory Health Research, University of Oulu, Oulu, Finland.

## Abstract

Globally, 41% of households, over 2.8 billion people, rely on solid fuels (coal and biomass) for cooking and heating. In developing countries in Asia and sub-Saharan Africa where these fuels are predominantly used, women who are customarily responsible for cooking, and their young children, are most exposed to the resulting air pollution. Solid fuels are still in widespread use and it appears that intervention efforts are not keeping pace with population growth in developing countries. Here we pinpoint the challenges and identify opportunities for addressing household air pollution while mitigating global climate change and promoting the sustainable development goals.

We recommend the following actions: implementation of the *WHO indoor air quality guidelines* *on household fuel combustion*; effective promotion and dissemination of improved cookstoves through formation of country alliances for clean cookstoves; expansion of liquefied petroleum gas production facilities and distribution networks; harnessing renewable energy potential; promotion of biogas production at both household and community level; ensuring improved ventilation of homes through education and enforcement of building standards; and exploiting opportunities in the health and other sectors for changing health-damaging cooking behaviour.

## Résumé

Dans le monde, 41% des foyers, à savoir plus de 2,8 milliards de personnes, utilisent des combustibles solides (charbon et biomasse) pour cuisiner et se chauffer. Dans les pays en développement d'Asie et d'Afrique subsaharienne, où ces combustibles sont majoritairement utilisés, les femmes, qui s'occupent habituellement de la cuisine, et les jeunes enfants sont les plus exposés à la pollution de l'air en résultant. Les combustibles solides sont encore très répandus et il apparaît que les actions menées ne progressent pas au même rythme que la croissance de la population dans les pays en développement. Nous identifions ici les difficultés et les possibilités de lutte contre la pollution de l'air domestique et, dans le même temps, les possibilités d'atténuation du changement climatique et de promotion des objectifs de développement durable.

Nous recommandons les actions suivantes: application des lignes directrices de l'OMS relatives à la qualité de l'air intérieur et à l'utilisation de combustibles dans les habitations; promotion et diffusion de modes de cuisson propres par la formation d'alliances pour des réchauds écologiques; développement de sites de production et de réseaux de distribution de gaz de pétrole liquéfié; exploitation du potentiel des énergies renouvelables; promotion de la production de biogaz au niveau des foyers et de la communauté; meilleure ventilation des logements à travers l'éducation et le respect des normes de construction; mise à profit des opportunités dans le secteur de la santé, entre autres, pour changer les comportements nuisibles à la santé lors de la préparation des repas.

## Resumen

El 41% de los hogares de todo el mundo, es decir, más de 2 800 millones de personas, depende de combustibles sólidos (carbón y biomasa) para la cocina y la calefacción. En países en desarrollo de Asia y del África subsahariana, en los cuales se utiliza principalmente este tipo de combustibles, las mujeres suelen ser las responsables de cocinar, por lo que sus hijos son los que más expuestos están a la contaminación del aire derivada de estas tareas. Los combustibles sólidos siguen utilizándose de forma generalizada y parece que los esfuerzos de intervención no están manteniendo el ritmo del crecimiento poblacional de los países en desarrollo. Aquí se detectan los problemas y se identifican las oportunidades para tratar la contaminación del aire en los hogares, a la vez que se mitiga el cambio climático global y se fomentan los objetivos de desarrollo sostenible.

Se recomienda tomar las siguientes medidas: la implementación de las normativas de la OMS para la calidad del aire interior en relación con los combustibles domésticos; el fomento y difusión eficaces de mejores cocinas mediante la formación de alianzas entre países para unas cocinas limpias; la expansión de instalaciones de producción de gas licuado del petróleo, tanto en los hogares como en las comunidades; el aprovechamiento potencial de las energías renovables; la promoción de la producción de biogás tanto en los hogares como en la comunidad; la garantía de una mejor ventilación en los hogares educando y fomentando los estándares de construcción; y el aprovechamiento de las oportunidades tanto en el sector sanitario como en otros sectores para cambiar el comportamiento perjudicial para la salud en las cocinas.

## ملخص

على الصعيد العالمي، تعتمد ‏41% من الأسر وأكثر من 2.8 مليار شخص على أنواع الوقود الصلب (الفحم والكتلة الحيوية) لأغراض الطهي والتدفئة. وفي البلدان النامية في آسيا والدول الإفريقية جنوب الصحراء الكبرى – حيث يشيع استخدام هذه الأنواع من الوقود – تتولى النساء عادةً مسؤولية الطهي مما يجعل أطفالهن الصغار الأكثر عرضة لتلوث الهواء الناتج عن الوقود. لا يزال استخدام الوقود الصلب شائعًا، ويبدو أن جهود التدخل لا تتواكب مع معدلات نمو السكان في البلدان النامية. ونحن نهدف هنا إلى الوقوف على التحديات وتمييز الفرص المتاحة لمعالجة تلوث الهواء المنزلي، بينما نسعى إلى التخفيف من آثار تغير المناخ العالمي وتعزيز أهداف التنمية المستدامة.

نحن نوصي بتنفيذ الإجراءات التالية: تنفيذ المبادئ التوجيهية الصادرة عن منظمة الصحة العالمية المعنية نوعية الهواء في الأماكن المغلقة فيما يتعلق باحتراق الوقود المنزلي؛ والترويج الفعال لمواقد الطهي المحسنة ونشرها من خلال تشكيل تحالفات بين البلدان لتوزيع مواقد الطهي النظيفة؛ وتوسيع مرافق إنتاج غاز النفط المسال وشبكات التوزيع؛ وتسخير إمكانات الطاقة المتجددة؛ وتعزيز إنتاج الغاز الحيوي على المستوى المجتمعي والمنزلي على حد سواء؛ وضمان تحسين التهوية في المنازل من خلال التعليم وتطبيق معايير البناء؛ واستغلال الفرص المتاحة في مجال الصحة والقطاعات الأخرى لتغيير سلوك الطهي المضر بالصحة.

## 摘要

综合来说，41% 的家庭，超过 28 亿人口利用固体燃料（煤炭和生物燃料）进行烹饪和取暖。在主要使用这些燃料的亚洲和撒哈拉以南非洲的发展中国家，女性通常负责烹饪，其年幼的孩子最容易受到其空气污染的影响。固体燃料目前仍广泛使用，并且貌似干预措施并未与发展中国家人口增长步伐一致。缓和全球气候变化并促进可持续发展目标时，我们将在此确定并明确解决家庭空气污染所面临的挑战和机会。

我们建议以下行为：实施世界卫生组织有关家庭燃料燃烧的室内空气质量指南；通过就洁净烹调用炉建立的国家联盟有效宣传并推广改进版烹调用炉；扩大液化石油气设备生产和经销网络；利用可再生能源的潜力；宣传家庭和社区使用生物燃气产品；确保通过教育改善家庭通风和强化建筑标准；并利用保健和其他部门的机会，从而改变对身体有害的烹饪行为。

## Резюме

Во всем мире в 41% домашних хозяйств свыше 2,8 млрд людей используют твердые виды топлива (уголь и биомассу) для приготовления пищи и отопления. В развивающихся странах Азии и Африки к югу от Сахары, где эти материалы применяются в качестве основного топлива, женщины, традиционно ответственные за приготовление пищи и заботящиеся о своих маленьких детях, больше других подвергаются влиянию загрязнения воздуха в результате своей деятельности. Твердое топливо по-прежнему широко используется, и, судя по всему, принятых мер недостаточно на фоне растущей численности населения в развивающихся странах. В данном документе делается акцент на проблемах загрязнения воздуха и определяются возможные пути их решения в домашних хозяйствах, минимизация воздействия на глобальное изменение климата и содействие достижению целей в области устойчивого развития.

Рекомендуется следующее: реализация руководящих принципов ВОЗ по обеспечению надлежащего качества воздуха в помещениях в части, относящейся к сжиганию топлива в домашних хозяйствах; эффективная популяризация и распространение усовершенствованных кухонных плит путем формирования на уровне страны объединений в поддержку чистых кухонных плит; развертывание объектов производства и сетей распространения сжиженного нефтяного газа; задействование потенциала возобновляемых источников энергии; содействие производству биогаза на уровне домашних хозяйств и общин; обеспечение улучшенной вентиляции жилищ путем образования и контроля соблюдения стандартов на строительство; использование возможностей в секторе здравоохранения и других секторах для изменения практик приготовления пищи, вредящих здоровью.

## Introduction

Globally, 41% of households, over 2.8 billion people, rely on solid fuels (coal and biomass) for cooking and heating.^1^ In developing countries, solid fuels are typically burnt in open fires and inefficient traditional cookstoves, often in poorly ventilated cooking spaces. Women who are customarily responsible for cooking, and their young children, are most exposed to the resulting high levels of air pollutants released including carbon monoxide (CO) and particulate matter (PM).

In 2010, household air pollution was estimated to be responsible for 3.5 million premature deaths worldwide.^2^ Household air pollution also contributes to outdoor air pollution, causing an additional 370 000 deaths and 9.9 million disability-adjusted life years globally in 2010.^3^ There is strong evidence linking household air pollution exposure with cardiovascular diseases,^4^^,^^5^ acute lower respiratory infections, chronic obstructive pulmonary disease and chronic bronchitis, lung cancer, cataract,^6^^,^^7^ low birth weight and stillbirth.^8^^,^^9^ Other health outcomes associated with household air pollution, for which evidence is less robust, include pharyngeal and laryngeal cancer,^10^^,^^11^ otitis media,^12^ asthma,^13^^,^^14^ tuberculosis,^15^ neonatal mortality^16^ and nutritional deficit.^17^ Indirect health effects from collecting firewood include assault of women and girls, insect (including disease vector) and snake bites, school absenteeism and musculoskeletal injuries from having to carry large bundles of firewood on the head and back for long distances.^18^

Solid fuels are still in widespread use in developing countries and it appears that intervention efforts are not keeping pace with population growth.^19^ The population mainly using solid fuel for cooking has remained unchanged over the last three decades at around 2.7 to 2.8 billion.^1^ Between 1980 and 2010, the population exposed to household air pollution increased from 333 million to 646 million in sub-Saharan Africa and from 162 million to 190 million in the eastern Mediterranean. In south-east Asia, the population exposed to household air pollution remained stable during the period at around 1 billion people.^1^

Recently, the World Health Organization (WHO) asserted that action to address the household air pollution problem has historically been slow, under-funded and ineffective.^20^ A systematic review of factors influencing uptake of cookstove interventions was recently published.^21^ Another review focusing on all interventions to reduce household air pollution and improve health in developing countries is in progress.^22^ Here we pinpoint the challenges, suggest improvements to existing interventions and identify new opportunities for addressing household air pollution in relation to the sustainable development goals (SDGs).^23^

### Indoor air quality guidelines

The recent *WHO indoor air quality guidelines*^20^ are tailored to the particular needs of developing countries where the burden of household air pollution is greatest. The guidelines recognize the challenges likely to be faced in implementation and provide detailed information on cookstove performance and potential health risks. Effective implementation of the guidelines will require strong environmental health programmes to improve understanding of the complexities of the household air pollution problem and inform national response.

### Improved cookstoves

Interventions to reduce household air pollution have primarily focused on the promotion and dissemination of improved cookstoves. However, despite the distribution of millions of improved cookstoves in developing countries over the last three decades, problems with household air pollution persist. This limited success is due to several factors, including lack of awareness of the problem and a lack of affordable stoves and fuels that reduce exposures appreciably.^24^^,^^25^ Lack of reliable exposure–response data has also been suggested as a reason for the failure of improved cookstoves to achieve the desired exposure reductions and health benefits.^26^

In China, the National Improved Stove Programme distributed about 130 million improved solid fuel stoves between 1980 and the early 1990s. However, household air pollution levels remained several times higher than national and WHO standards.^27^ In India, the National Programme for Improved Chulhas (traditional stoves) distributed more than 30 million improved stoves between 1985 and 2002. This programme was also widely regarded as a failure due to poor uptake and high air pollution emission levels.^28^^–^^30^ Recent evaluations of interventions to promote improved cookstoves in south Asia have also revealed that their purported benefits may not have been realized.^31^^,^^32^ When stove interventions are well designed, implemented and monitored, they can have positive effects, but are unlikely to reduce household air pollution to levels recommended by WHO.^33^ The challenge therefore is to design fuel efficient stoves that reduce emissions to levels that are low enough to translate into health benefits.

Advanced combustion biomass stoves show substantial emissions’ reductions over traditional stoves, but cannot yet match emission levels from liquefied petroleum gas (LPG).^34^ In many countries, including China, Ethiopia, Ghana, India, Kenya and Sri Lanka, locally manufactured stoves, which are usually cheap, dominate the market. These stoves are fuel efficient, but still have high air pollution emissions. Many past stove programmes and even some current programmes distribute cookstoves built by local artisans.^35^ Data from both laboratory and field settings suggest many of the stoves currently on the market are effectively fuel saving but have limited benefit in terms of emissions.^36^

Since 2010, the Global Alliance for Clean Cookstoves (GACC) has led global efforts through engagement of interest groups including government ministries and agencies, manufacturers, distributors and users. Their goal is to switch 100 million households to clean cookstoves by 2020. Standardizing the testing of cookstoves is important to ensure that only fuel efficient cookstoves which also lower emissions are adopted. This requires product standards’ agencies in countries to be adequately resourced and empowered. The International Organization for Standardization (ISO) provides guidelines for evaluating cookstove performance in terms of fuel efficiency, total emissions (CO and PM_2.5_), indoor emissions (CO and PM_2.5_) and safety (International Workshop Agreement (IWA) 11:2012). These guidelines are currently being developed into formal ISO standards that will lead to certification of cookstoves and other clean cooking devices (ISO Technical Committee 285).

Sustained use of improved cookstoves is impeded by cultural issues and other factors such as cooking in traditional utensils, multiple and bulk cooking, prolonged cooking time, poor stove design and the need for frequent maintenance.^37^ This situation often leads to stove stacking (the use of multiple stoves at one time). Design considerations, time saving and suitability for cooking traditional dishes have been mentioned as enablers of household uptake of improved cookstoves.^21^

Improved stoves will not necessarily be accepted by households^38^ unless stoves are designed to be compatible with the shapes of traditional cooking pots and modes of preparation of traditional foods. Evidence from multiple settings suggests that some clean and efficient cookstoves are not designed to execute the desired cooking tasks; this leads to continued use of traditional cookstoves alongside the improved stoves.^39^^–^^44^ Successful implementation of cookstove programmes requires the involvement of women in designing the stoves, the training of users and follow-up in communities to address concerns.^24^^,^^34^

### Liquefied petroleum gas

LPG is clean, burns efficiently, is easy to use, reduces cooking time and can significantly reduce emissions. To date, only one study conducted in Sudan has evaluated the impact of LPG use on household air pollution levels. This study reported substantial reductions in kitchen PM (51–80%) and CO (74–80%) levels.^45^ GACC-funded trials are presently underway in Ghana and Nepal to evaluate the impact of LPG and other clean cooking interventions on child survival outcomes. These trials will provide further evidence on the feasibility of LPG usage for reducing household air pollution and associated health risks.

Poverty and supply chain issues are major barriers to adoption of LPG for cooking in developing countries. LPG is expensive and may not be readily available due to limited distribution networks and competing use in motor vehicles. The limited distribution networks mean household members have to travel long distances to purchase the product, presenting additional cost to the household. The start-up cost (purchase of cooker, cylinder, regulator and hose) for using LPG at home is too high for most low-income households.^46^ Expanding LPG production facilities and distribution networks in developing countries requires a major financial commitment and often private sector involvement. The Global LPG Partnership aims to help developing countries overcome barriers to the widespread use of LPG through provision of capital and knowledge to expand LPG supply, infrastructure and distribution systems; assistance with policy and regulatory reforms to attract foreign investors; and financing LPG usage start-up costs. The World LPG Association has a key goal to inform and educate all stakeholders about the benefits of LPG and is committed to rolling out clean energy in developing countries.

Because LPG is heavily subsidized in many countries to promote household use, commercial vehicle owners have exploited the situation by refitting their vehicles to use LPG. This problem can be solved by creating two market prices for LPG (a subsidized price for domestic users and an unsubsidized price for vehicle users) or by legislating against retrofitting vehicles to use LPG. Although LPG subsidies have helped to make the product more accessible, the subsidized price is still beyond the reach of many low-income households.^47^ Social protection programmes in these countries should consider the provision of LPG.

### Renewable energy resources

Solar, wind, hydro and geothermal power can serve as safe, affordable sources of household energy while mitigating global climate change.^48^^,^^49^ Most countries have renewable energy potential many times their current energy consumption that can be exploited with current technology.^50^ For example, many areas of sub-Saharan Africa experience daily solar radiation of between 14.4 and 21.6 MJ/m^2^.^51^ Geothermal resources are abundant in east Africa^52^ with great potential for wind power also present around the coastal regions and eastern highlands.^53^ The Green Climate Fund^54^ is a promising source of funds to develop the infrastructure required to exploit these renewable energy resources.

Biogas, produced from the breakdown of biodegradable materials under anaerobic conditions, also has the potential to reduce dependence on solid fuels in developing countries. Developing countries are beset with numerous waste management problems. Municipal and human wastes, which pose environmental and human health threats if not well managed, can instead serve as feedstock for biogas production. Biogas production can also reduce greenhouse gas emissions and improve livelihoods and health.^49^ China has about 750 large- and medium-scale industrial biogas plants installed, over 7.5 million biogas digesters in use in households and a network of rural biogas service centres.^55^ India also has a large household-scale programme with active programmes also found in Kenya, Nepal, Sri Lanka and several countries in Latin America.^55^ Bio-latrines, a low maintenance system, can replace pit latrines which are in widespread use in developing countries (Box 1). Methane gas produced by anaerobic decomposition of the sewage is collected and stored for domestic use. The treated waste is high in plant nutrients and can be used or sold as fertilizer to generate income. However, bio-latrines are not always culturally acceptable.

Box 1 **The triple dividend of bio-latrine technology in Kitale, western Kenya: improved sanitation, clean cooking and lighting and fertilizer for farming**

Kitale is a small agricultural town located in western Kenya with a rapidly growing population (106 187 in 2009). Almost two thirds of the town’s residents live in slums and informal settlements which are water-logged and have no piped water, sewers or sanitation services. Water is thus sourced from the river in the area which is contaminated with sewage, oil and solid waste. Sanitation in these informal settlements is very poor and is driven by the lack of affordable sanitation options, low awareness of potential health hazards and land tenure insecurity. The situation has resulted in several adverse health outcomes including high levels of waterborne diseases and ill-health.

Bio-latrines have been installed in four primary schools in the informal settlements and provide safe and hygienic sanitation facilities for 2780 children. The bio-latrines also provide fuel for cooking in the school kitchens and lighting in the classrooms to enable the pupils, especially those residing in crowded single occupancy rooms, to study in the evenings. The bio-latrines further generate organic fertilizer for use in the school farms to support the school meals programme. Local manufacturers have quickly realized the potential of the bio-latrine technology and have become involved in the construction of bio-latrines in the community.

Source: Khatavkar and Matthews.^56^

### Housing improvements

Housing improvements and modifications also offer potential for significantly reducing household air pollution exposure. Creating and enlarging kitchen windows, fitting flues and smoke hoods, enlarging roof spaces, raising cooking surfaces from ground level to waist height and separating cooking areas from other living spaces are important modifications that should be promoted. Education and information dissemination have traditionally been the approach to ensuring housing improvements for improved health. In developing countries, this approach has failed and the key to the success of this strategy is enforcement of building standards. Unfortunately, in low-income countries, enforcing building standards is also a major challenge, as construction is often informal without plans and permits. Building inspectorate departments need to be better resourced, to enable them carry out their functions efficiently.

### Behavioural change

A recent review of behavioural change interventions to reduce childhood household air pollution exposure reported that behavioural change strategies have the potential to reduce household air pollution exposure by 20–98% in laboratory settings and 31–94% in field settings.^57^ Household air pollution exposure can be reduced by cooking outdoors, reducing time spent in the cooking area, keeping the kitchen door open while cooking, avoiding leaning over the fire while attending to the cooking, avoiding carrying children while cooking and keeping children away from the cooking area. Opportunities to educate communities on reducing household air pollution exposure include durbars, festival celebrations, religious meetings and child welfare outreach clinics. Community health workers are the fulcrum of the health system in many developing countries^58^ and represent an excellent resource for educating communities.

The GACC and future country alliances should engage national health authorities to incorporate household air pollution and clean cooking in the training modules of frontline health workers. Community-based health surveillance volunteers assist with health service delivery in some Sub-Saharan African countries, notably Ethiopia, Ghana, Mali and Niger.^59^ In other countries, environmental health and sanitation officers of local government departments are responsible for ensuring proper environmental and sanitary conditions in communities. These people are other potential resources for educating communities.

## Recommendations

Actions to reduce household air pollution in developing countries should also help to achieve important SDG targets (Table 1). Implementation of the *WHO indoor air quality guidelines* *on household fuel combustion* is strongly recommended and requires WHO to provide strong technical support to countries through their regional and country offices. This will help achieve a very important health-related SDG target (3.9). It is within the mandates of environmental protection agencies in these countries to lead the implementation process but the involvement of all stakeholders, including communities, and academic and research institutions, is required. Governments should endeavour to adequately resource these agencies to effectively take up the task, and in countries where no such agencies exist, they should be supported by development partners to establish an agency.

**Table 1:** Recommended actions for reducing household air pollution and implications for the Sustainable Development Goals

Sustainable Development Goal and Targets**^23^**	Recommended action
**3: Ensure healthy lives and promote well-being for all at all ages**	
3.9: By 2030, substantially reduce the number of deaths and illnesses from hazardous chemicals and air, water and soil pollution and contamination	Implementation of *WHO indoor air quality guidelines on household fuel combustion* Housing improvements and modifications through education and enforcement of building standards Behavioural change through education at community meetings and outreach points
**6: Ensure availability and sustainable management of water and sanitation for all**	
6.2: By 2030, achieve access to adequate and equitable sanitation and hygiene for all and end open defecation, paying special attention to the needs of women and girls and those in vulnerable situations	Promotion of biogas production at both household and community level
6.3: By 2030, improve water quality by reducing pollution, eliminating dumping and minimizing release of hazardous chemicals and materials, halving the proportion of untreated wastewater and increasing recycling and safe reuse globally	
**7: Ensure access to affordable, reliable, sustainable and** **modern energy for all**	
7.1: By 2030, ensure universal access to affordable, reliable and modern energy services 7.b: By 2030, expand infrastructure and upgrade technology for supplying modern and sustainable energy services for all in developing countries, in particular least developed countries and small island developing states	Expansion of liquefied petroleum gas production facilities and distribution networks
7.2: By 2030, increase substantially the share of renewable energy in the global energy mix 7.a: By 2030, enhance international cooperation to facilitate access to clean energy research and technology, including renewable energy, energy efficiency and advanced and cleaner fossil-fuel technology, and promote investment in energy infrastructure and clean energy technology	Investment in renewable energy technology
**11: Make cities and human settlements inclusive, safe, resilient and sustainable**	
11.1: By 2030, ensure access for all to adequate, safe and affordable housing and basic services and upgrade slums	Housing improvements and modifications through education and enforcement of building standards
11.6: By 2030, reduce the adverse per capita environmental impact of cities, including by paying special attention to air quality and municipal and other waste management	Promotion of biogas production at both household and community level
**12: Ensure sustainable consumption and production patterns**	
12.5: By 2030, substantially reduce waste generation through prevention, reduction, recycling and reuse	Promotion of biogas production at both household and community level
12.a: Support developing countries to strengthen their scientific and technological capacity to move towards more sustainable patterns of consumption and production	Investment in renewable energy technology
**13: Take urgent action to combat climate change and its impacts**	
13.a: Implement the commitment undertaken by developed-country parties to the United Nations Framework Convention on Climate Change to a goal of mobilizing jointly US$100 billion annually by 2020 from all sources to address the needs of developing countries in the context of meaningful mitigation actions and transparency on implementation and fully operationalize the Green Climate Fund through its capitalization as soon as possible	Investment in renewable energy technology
**15: Protect, restore and promote sustainable use of terrestrial ecosystems, sustainably manage forests, combat desertification, and halt and reverse land degradation and halt biodiversity loss**	
15.2: By 2020, promote the implementation of sustainable management of all types of forests, halt deforestation, restore degraded forests and increase afforestation and reforestation globally	Effective promotion and dissemination of improved cookstoves

US$: United States Dollars, WHO: World Health Organization.

Ensuring improved ventilation of homes through education of communities on the health benefits and enforcement of building standards is also required; local government authorities are responsible for implementing this recommendation. Countries should also consider exploiting opportunities in health and other sectors, and communities, to change health-damaging cooking behaviour of households.

We recommend building biogas plants in metropolitan areas especially, where the feedstock seems readily available due to the mounting waste management problems in these areas, and promoting bio-latrine technology in rural areas where they are culturally acceptable. Implementing this recommendation requires collaboration between energy ministries and local government authorities in the countries concerned and will drive water and sanitation targets (SDG 6.2, 6.3 and 12.5). Governments should also seek technical and financial assistance, both locally and externally, to expand LPG production facilities and distribution networks and to harness their renewable energy potential. These actions will help achieve important sustainable energy and consumption, and climate change targets (SDG 7.1, 7.2, 7.a, 7.b, 12.a and 13.a).

Finally, effective promotion and dissemination of improved cookstoves is also recommended. This requires the formation of country alliances for clean cookstoves to seek the engagement of all stakeholders including manufacturers and users and provide a platform for sharing ideas, addressing concerns and collectively setting sector-wide goals and targets. An important forest conservation target (SDG 15.2) will be promoted through implementation of this recommendation.

## Conclusion

Solid fuels are still in widespread use in developing countries and it appears that intervention efforts are not achieving their desired goals. Providing clean household energy solutions in the effort to tackle household air pollution in developing countries can also mitigate global climate change and help to achieve several of the sustainable development goals.
